# Editorial: Neurofeedback in ADHD

**DOI:** 10.3389/fnhum.2015.00602

**Published:** 2015-10-30

**Authors:** Martijn Arns, Hartmut Heinrich, Tomas Ros, Aribert Rothenberger, Ute Strehl

**Affiliations:** ^1^Research Institute BrainclinicsNijmegen, Netherlands; ^2^Department of Experimental Psychology, Utrecht UniversityUtrecht, Netherlands; ^3^Department of Child and Adolescent Mental Health, University Hospital of ErlangenErlangen, Germany; ^4^kbo-Heckscher-KlinikumMünchen, Germany; ^5^Laboratory for Neurology and Imaging of Cognition, Department of Neurosciences, University of GenevaGeneva, Switzerland; ^6^Child and Adolescent Psychiatry, University Medical Center GöttingenGöttingen, Germany; ^7^Institute of Medical Psychology and Behavioral Neurobiology, University of TuebingenTuebingen, Germany

**Keywords:** neurofeedback, ADHD, operant conditioning, classical conditioning, EEG

Almost a century ago Ivan Pavlov laid the groundwork for what we now know as classical conditioning. Not long after this first description of classical conditioning, and the first description of the human EEG by Berger (1929), early observations were made that the human EEG (alpha blocking response) could be classically conditioned (Durup and Fessard, 1935; Loomis et al., 1936). This alpha blocking response consists of a desynchronization of the dominant alpha activity, present during an eyes closed (or dark) condition, into a desynchronized low voltage beta EEG (also see Ros et al., 2014, in this research topic). More systematic studies demonstrated that the alpha blocking response fulfilled all of the Pavlovian types of conditioning (Jasper and Shagass, 1941a) and could not be explained by sensitization (Knott and Henry, 1941). Jasper and Shagass took their experiments one step further, showing that using these principles of conditioning, subjects could be taught “voluntary control” over their alpha blocking response, by pairing the light-onset not to an auditory tone, but to a sub-vocal command (“block”; Jasper and Shagass, 1941b). In their most basic form, these can be considered the first demonstrations of “neurofeedback” or voluntary control over the EEG based on basic learning principles. Some years after these initial studies, the first reports employing operant learning principles to EEG were reported by Kamiya [voluntary control of alpha power and alpha peak frequency (Kamiya, 1968)], McAdam et al. [voluntary control of the contingent negative variation (CNV) or slow cortical potential (SCP) (McAdam et al., 1966)], and Sterman (operant conditioning of the so-called sensori-motor rhythm (SMR) in cats, Wyrwicka and Sterman, 1968). Interestingly, from a historical perspective, these EEG parameters are still the focus of intensive study in neurofeedback research, as this research topic nicely illustrates.

Neurofeedback as a therapeutic intervention has been most comprehensively investigated for the treatment of attention-deficit/hyperactivity disorder (ADHD), in line with the theme of this research topic. Leading from a review by Albrecht et al. (2015) on the neurophysiological background of this child psychiatric disorder, including its comorbidities, the efficacy of neurofeedback in the treatment of ADHD is discussed in great detail. The current controversy regarding the efficacy of neurofeedback in ADHD is centered on the fundamental question of how it should be evaluated: namely, in accordance with the APA guidelines (used to evaluate psychological treatments), or along the lines of drug treatments (requiring double-blind placebo controlled designs). In their perspective article, Vollebregt et al. (2014) review this issue in more detail, alongside Gevensleben and colleagues who investigated the feasibility of a double-blind placebo controlled design for SCP neurofeedback (Gevensleben et al., 2014a). A further interesting approach was undertaken by Micoulaud-Franchi and colleagues, who report an updated meta-analysis of neurofeedback studies in ADHD (Micoulaud-Franchi et al., 2014). Using a comparable approach as the European ADHD Guidelines group (Sonuga-Barke et al., 2013), they demonstrated significant small to medium effect sizes specifically for inattention, in line with an earlier meta-analysis that also revealed strongest effects for the same domain (Arns et al., 2009). In addition, Christiansen and colleagues report preliminary results of a randomized controlled trial comparing SCP neurofeedback to a self-management program (Christiansen et al., 2014).

As is clear from the historical studies mentioned above, neurofeedback is built on the foundations of learning theory. Therefore, it is crucial to dissociate “neurofeedback as a treatment” from “neurofeedback as entertainment.” The “neurofeedback as entertainment” is an approach popularized by many modern devices such as the Mattel Mindflex (keep a ball in the air using your brain activity) or consumer-grade EEG units such as the Emotiv Epoc which run brain-training “apps.” In the same way as there is a difference between “reading a book” for entertainment purposes and “studying a book” to learn how to apply a specific technique it is no different for neurofeedback. Unfortunately in some clinical studies the goal has been to “entertain” children with “EEG-driven games,” rather than really applying a learning procedure the children could benefit from for a longer period. In this respect, the contributions from Strehl (2014) and Zuberer et al. (2015) are important and valuable contributions covering aspects of learning theory. Gevensleben and colleagues additionally discuss different neurocognitive models of how neurofeedback works (Gevensleben et al., 2014b). Ros and colleagues go one step further by offering a firmly neurophysiological account, proposing a “systems neuroscience framework” for tuning pathological brain oscillations (Ros et al., 2014).

Up to now, most neurofeedback protocols in the treatment of ADHD (e.g., SMR, Theta/Beta, and SCP Feedback) have shown comparable effect sizes on ADHD domains such as inattention, impulsivity and hyperactivity (reviewed in Arns et al., 2014b). In this research topic further indications for specificity of various neurofeedback protocols emerge. Studer and colleagues originally report protocol specific effects on motor system excitability, as well as P3 amplitudes and CNV amplitudes for Theta/Beta and SCP neurofeedback (Studer et al., 2014). Arns and colleagues further reveal that although clinically both SMR and Theta/Beta neurofeedback have similar effects, only for SMR neurofeedback the clinical effects are mediated by a normalization of sleep-onset latency, suggesting the clinical effects of Theta/Beta neurofeedback are mediated via a different mechanism (Arns et al., 2014a).

Although the majority of current research has utilized neurofeedback protocols that stem from before the twenty-first century, it is also important to look ahead and acknowledge new developments. With respect to individualized treatment, it may be adequate to adapt protocols as suggested by an EEG study of attention in Heinrich et al. (2014), and the theoretical framework of Ros et al. (2014). Several contributions also introduce new and promising approaches to neurofeedback, such as the contribution by Marx and colleagues, who compared SCP neurofeedback with Near Infrared Spectroscopy (NIRS) neurofeedback in children with ADHD, providing feedback from a signal physiologically similar to the fMRI BOLD response (Marx et al., 2014). Also, the perspective article by Bauer and Pllana provides further insights and opportunities in the application of EEG-based tomographic neurofeedback, theoretically enabling feedback of more focal brain activity (Bauer and Pllana, 2014).

We hope that you will enjoy this research topic, study and apply it in practice, unless you read it only for entertainment purposes!

## Conflict of interest statement

The authors declare that the research was conducted in the absence of any commercial or financial relationships that could be construed as a potential conflict of interest.
